# Association of the preoperative triglyceride-glucose index with postoperative atrial fibrillation following esophagectomy: a retrospective cohort study with a focus on non-diabetic patients

**DOI:** 10.3389/fnut.2026.1785056

**Published:** 2026-05-29

**Authors:** Yang Gao, Haijun Sun, Xiuyu Wu, Xinkui Xiong, Chao Liu, Qingquan Wu, Jianqiang Zhao, Zhiwei Xu, Hui Xia, Zhiyun Xu

**Affiliations:** 1 Department of Cardiothoracic Surgery, The Affiliated Huai'an No. 1 People's Hospital of Nanjing Medical University, Huai'an, China; 2 Department of Thoracic Surgery, The First People's Hospital of Lianyungang City, Nanjing Medical University Affiliated Lianyungang Clinical College, Lianyungang, China; 3 School of Clinical Medicine, Nanjing Medical University, Nanjing, China; 4 Department of Anesthesiology, The Affiliated Huai'an No. 1 People's Hospital of Nanjing Medical University, Huai'an, China

**Keywords:** esophagectomy, internal validation, non-diabetic patients, postoperative atrial fibrillation, triglyceride-glucose index, TyG index

## Abstract

**Background:**

Postoperative atrial fibrillation (POAF) is common after esophagectomy. The triglyceride-glucose (TyG) index is an accessible cardiometabolic marker derived from fasting triglyceride and glucose levels, but its relationship with POAF after esophagectomy, especially in patients without diabetes mellitus, remains unclear. We evaluated the association between preoperative TyG and POAF and assessed whether TyG added information beyond selected clinical risk factors.

**Methods:**

This single-center retrospective cohort study screened consecutive adults who underwent curative esophagectomy for histologically confirmed esophageal squamous cell carcinoma between January 2022 and April 2026. Patients receiving preoperative neoadjuvant therapy were excluded. TyG was calculated from preoperative fasting triglyceride and glucose values. The primary outcome was new-onset POAF within 7 postoperative days. Reduced multivariable logistic models were constructed according to event-per-variable considerations. Bayesian logistic regression, restricted cubic spline analysis, ROC analysis, bootstrap internal validation, calibration assessment, and incremental value analyses were performed. A sensitivity analysis was conducted in patients without diabetes.

**Results:**

Among 554 patients, 76 developed POAF. In the reduced model adjusted for age, sex, diabetes mellitus, and left atrial diameter, higher TyG was associated with increased odds of POAF (OR 4.02, 95% CI 1.90–8.48; *P* < 0.001). Bayesian analysis was consistent (posterior OR 3.31, 95% credible interval 1.64–6.70). Adding TyG to the baseline clinical model increased apparent AUC from 0.667 to 0.716 and optimism-corrected AUC from 0.647 to 0.694; continuous NRI and IDI were 0.441 and 0.024, respectively. Spline analysis showed a positive overall association without statistically significant non-linearity. In the non-diabetic subgroup (*n* = 489), TyG remained associated with POAF (adjusted OR 3.65, 95% CI 1.66–8.02; *P* = 0.001), with consistent Bayesian and validation findings.

**Conclusion:**

Higher preoperative TyG was associated with POAF after esophagectomy, including among patients without diabetes. TyG provided modest incremental predictive information beyond selected clinical factors, but these exploratory findings require external validation before clinical thresholds or TyG-guided interventions can be recommended.

## 1 Introduction

Esophageal cancer remains one of the most aggressive malignancies worldwide, and esophagectomy continues to be a cornerstone of curative treatment for resectable disease (1). Despite advances in surgical techniques, anesthesia, and perioperative care, postoperative atrial fibrillation (POAF) remains a frequent complication after esophagectomy, with reported incidences ranging from approximately 10% to 30% (2). Although POAF may be transient in some patients, it has been associated with hemodynamic instability, thromboembolic events, prolonged intensive care and hospital stay, and adverse postoperative outcomes (3, 4). Given the substantial physiological stress imposed by esophagectomy, identifying simple and clinically accessible preoperative markers that may help refine POAF risk assessment remains important (5).

The pathophysiology of POAF is complex and multifactorial. Surgical trauma, systemic inflammation, oxidative stress, autonomic imbalance, fluid shifts, and pre-existing atrial structural or electrical vulnerability may all contribute to postoperative atrial arrhythmogenesis (6, 7). In addition to these established perioperative and cardiac factors, cardiometabolic status has attracted increasing attention as a potential contributor to atrial arrhythmia risk (8). Abnormal glucose-lipid metabolism may be associated with endothelial dysfunction, inflammatory activation, and myocardial metabolic vulnerability, thereby potentially lowering the threshold for atrial fibrillation under perioperative stress (9). However, direct assessment of insulin resistance using insulin-based methods, such as the hyperinsulinemic-euglycemic clamp or the homeostatic model assessment of insulin resistance, is not routinely feasible in surgical practice.

The triglyceride-glucose (TyG) index, calculated from fasting triglyceride and fasting plasma glucose levels, has been widely used in clinical and epidemiological studies as a convenient cardiometabolic marker and a practical surrogate indicator of insulin resistance (10). Because both triglyceride and glucose measurements are routinely obtained during preoperative evaluation, the TyG index is inexpensive, reproducible, and readily available in real-world perioperative settings. Previous studies have linked a higher TyG index to adverse cardiovascular outcomes and atrial fibrillation in non-surgical populations, and its potential prognostic value has also been explored in selected perioperative settings (11). Nevertheless, whether the preoperative TyG index is associated with POAF after esophagectomy remains insufficiently characterized. This question is clinically relevant because the perioperative context of esophagectomy differs substantially from community-based cardiovascular cohorts and cardiac surgery populations, involving major thoracic surgical trauma, autonomic disturbance, inflammatory stress, and nutritional vulnerability (12).

An additional issue is whether the TyG index retains clinical relevance among patients without diagnosed diabetes mellitus. Diabetes is a recognized risk factor for atrial fibrillation, but diabetes status alone may not fully capture the spectrum of cardiometabolic heterogeneity. Some patients without overt diabetes may still have unfavorable glucose-lipid profiles or subclinical metabolic abnormalities that are not reflected by diagnostic categories alone (13). Therefore, evaluating the association between TyG and POAF in non-diabetic patients may help clarify whether this readily available marker provides information beyond conventional diabetes status (14). In addition, the shape of the dose-response relationship between TyG and POAF, as well as the incremental value of adding TyG to selected clinical risk factors, remains uncertain.

Therefore, in this retrospective cohort study of patients undergoing curative esophagectomy for esophageal squamous cell carcinoma, we aimed to evaluate the association between the preoperative TyG index and POAF. We further sought to examine the dose-response pattern using restricted cubic spline analysis, assess whether the association persisted among patients without diabetes mellitus, and determine whether TyG provided incremental predictive information beyond selected clinical factors.

## 2 Methods

### 2.1 Study design and participants

**Figure 1:**
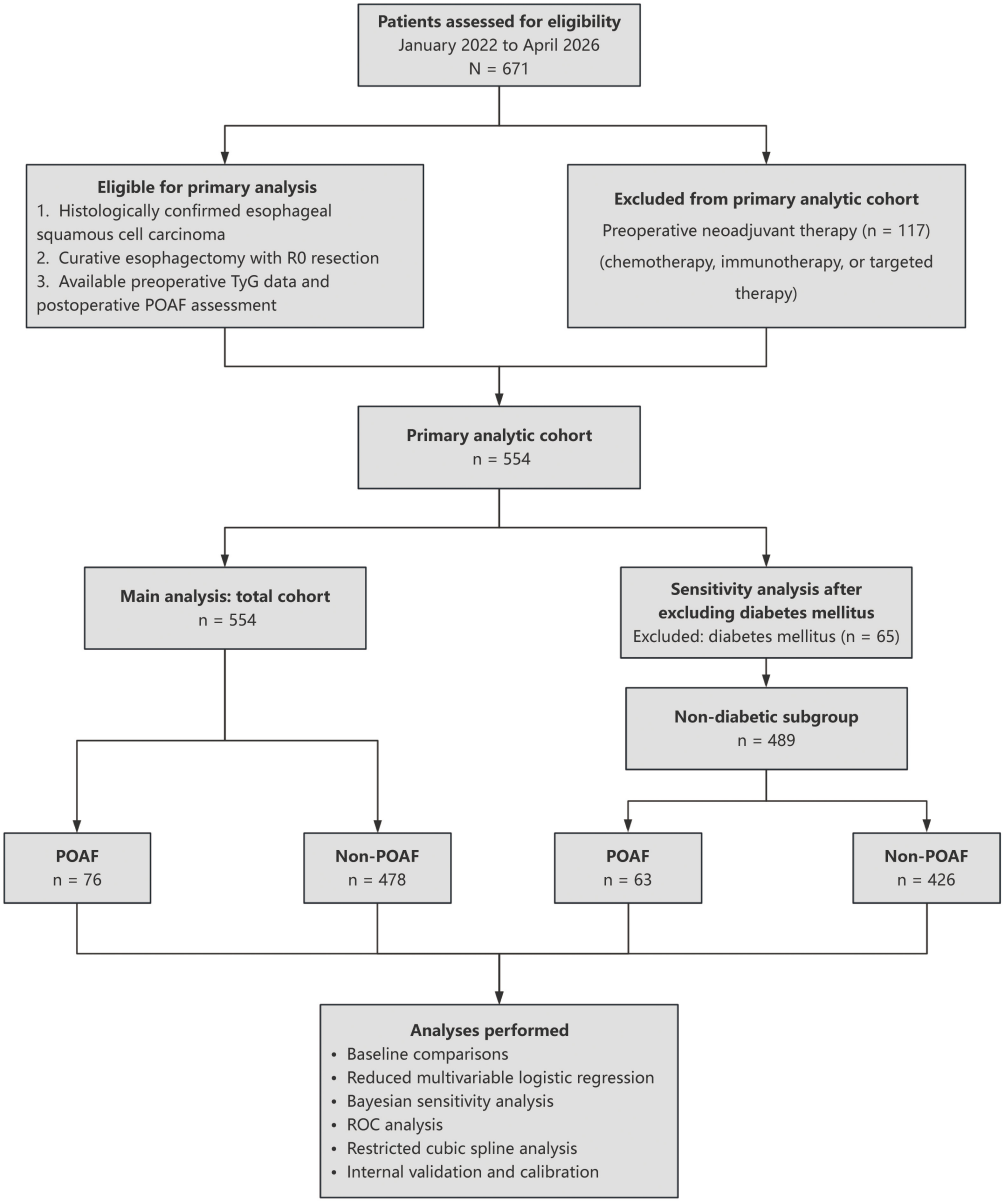
Flow diagram of the study population. A total of 671 consecutive patients who underwent curative esophagectomy for histologically confirmed esophageal squamous cell carcinoma between January 2022 and April 2026 were assessed for eligibility. After excluding 117 patients who received preoperative neoadjuvant therapy, 554 patients were included in the primary analytic cohort, including 76 with POAF and 478 without POAF. After excluding 65 patients with diabetes mellitus, 489 patients were included in the non-diabetic subgroup, including 63 with POAF and 426 without POAF.

This retrospective cohort study was conducted at The Affiliated Huai'an No. 1 People's Hospital of Nanjing Medical University. Consecutive adult patients who underwent curative esophagectomy for histologically confirmed esophageal squamous cell carcinoma between January 2022 and April 2026 were screened for eligibility. This study was reported in accordance with the Strengthening the Reporting of Observational Studies in Epidemiology (STROBE) statement. Patients were eligible for the primary analysis if they met the following criteria: (1) histologically confirmed esophageal squamous cell carcinoma; (2) curative esophagectomy with R0 resection; and (3) available preoperative triglyceride-glucose (TyG) index data and postoperative rhythm assessment sufficient to ascertain postoperative atrial fibrillation (POAF). Patients who received preoperative neoadjuvant therapy, including chemotherapy, immunotherapy, or targeted therapy, were excluded from the primary analytic cohort. This exclusion criterion was applied because preoperative antitumor therapy may substantially influence tumor stage, nutritional status, systemic inflammation, glucose-lipid metabolism, and perioperative risk, thereby introducing additional clinical heterogeneity. Preoperative atrial arrhythmia history was assessed by reviewing available medical records, admission history, previous diagnoses, and preoperative electrocardiographic data. Patients with documented preoperative atrial fibrillation or atrial flutter were not considered to have incident POAF. Because of the retrospective design, asymptomatic or undocumented paroxysmal atrial fibrillation before surgery could not be completely excluded. To evaluate the potential selection bias introduced by excluding patients who received neoadjuvant therapy, baseline characteristics and POAF incidence were compared between the included analytic cohort and patients excluded because of neoadjuvant therapy. A total of 671 patients were assessed for eligibility. After excluding 117 patients who received preoperative neoadjuvant therapy, 554 patients were included in the primary analytic cohort. For the sensitivity analysis focused on patients without diabetes mellitus, 65 patients with diabetes mellitus were excluded, leaving 489 patients in the non-diabetic subgroup. The participant selection process is summarized in Figure 1. The study protocol was approved by the Institutional Ethics Committee of Nanjing Medical University (Reference No. KY-2026-072-01). The study was conducted in accordance with the Declaration of Helsinki. Owing to the retrospective design and the use of de-identified clinical data, the requirement for written informed consent was waived by the Ethics Committee.

### 2.2 Exposure assessment: triglyceride-glucose index

The primary exposure was the preoperative TyG index. The TyG index was calculated using fasting triglyceride and fasting plasma glucose measurements obtained before surgery. When more than one eligible preoperative measurement was available, the values closest to the date of surgery were used. Whenever possible, triglyceride and glucose values obtained from the same blood draw were used; when this was unavailable, fasting measurements obtained on the same day were used. The TyG index was calculated as follows: TyG index = ln [fasting triglycerides (mg/dL) × fasting plasma glucose (mg/dL) / 2]. When triglyceride and/or glucose values were reported in mmol/L, they were converted to mg/dL before calculation using the following conversion factors: triglycerides, mmol/L × 88.57; glucose, mmol/L × 18. Fasting status was considered confirmed when overnight fasting was documented in the laboratory order, nursing record, or preoperative medical record. Because fasting status could not be verified in all patients, fasting status confirmation was collected and reported as a baseline variable. Preoperative statin and metformin use were also extracted from medication records because these medications may influence lipid or glucose metabolism. The TyG index was analyzed as a continuous variable in regression models and restricted cubic spline analyses. No primary clinical cut-off value for TyG was prespecified, and cut-off-based decision-making was not pursued in the main analysis.

### 2.3 Outcome definition: postoperative atrial fibrillation

The primary outcome was POAF, defined as new-onset atrial fibrillation or atrial flutter occurring within 7 days after esophagectomy in patients without documented preoperative atrial fibrillation or atrial flutter. All patients underwent continuous rhythm monitoring during the first 72 postoperative hours in the intensive care unit. From postoperative day 4 to postoperative day 7, POAF events were identified through routine postoperative surveillance in the ward, 12-lead electrocardiography when clinically indicated, and review of medical and nursing records, including cardiology consultation notes and treatment documentation. POAF was considered present if a new-onset atrial fibrillation or atrial flutter episode lasted at least 30 s. Episodes were also classified as POAF when the exact duration was not available in the medical record but the arrhythmia was documented by electrocardiography and required medical intervention, including pharmacological rate control, rhythm control, or electrical cardioversion.

### 2.4 Covariates

Baseline demographic, clinical, laboratory, medication, nutritional, oncological, echocardiographic, and perioperative variables were extracted from the hospital electronic medical record system. Demographic and lifestyle variables included age, sex, body mass index (BMI), smoking status, and drinking status. Comorbidities included hypertension, diabetes mellitus, and coronary heart disease. Diabetes mellitus and other comorbidities were identified from documented diagnoses in the medical record and medication history. Medication variables included preoperative statin use and metformin use. Nutritional indicators included serum albumin and percentage weight loss within 3 months before surgery. Tumor-related information included clinical TNM stage abstracted from the preoperative staging record. Cardiac structure was assessed using preoperative transthoracic echocardiography, with left atrial diameter used as the primary echocardiographic variable because of its established clinical relevance to atrial arrhythmia risk. Perioperative variables included operative duration and estimated intraoperative blood loss. The covariates collected for descriptive and exploratory analyses were broader than those included in the final multivariable models. To reduce the risk of overfitting, the final multivariable models were intentionally restricted to a small number of clinically relevant variables according to event-per-variable considerations. Therefore, variables such as BMI, smoking, drinking, hypertension, coronary heart disease, fasting status confirmation, statin use, metformin use, albumin, weight loss, clinical TNM stage, operative duration, and intraoperative blood loss were described and explored but were not all entered into the main reduced multivariable models.

### 2.5 Statistical analysis

Continuous variables were presented as mean ± standard deviation or median with interquartile range, as appropriate. Categorical variables were presented as number and percentage. Between-group comparisons were performed using the independent-samples *t* test or Mann–Whitney U test for continuous variables and the chi-square test or Fisher's exact test for categorical variables, as appropriate. First, baseline characteristics were compared between patients included in the primary analytic cohort and patients excluded because of preoperative neoadjuvant therapy. This comparison was performed to assess the potential selection bias associated with excluding patients who received preoperative chemotherapy, immunotherapy, or targeted therapy. Baseline characteristics were also compared between patients with and without POAF in the total cohort and in the non-diabetic subgroup. Univariable logistic regression analyses were performed to examine crude associations between candidate variables and POAF. Odds ratios (ORs) and 95% confidence intervals (CIs) were reported. Univariable analyses were considered exploratory and were used to describe the association pattern of candidate variables rather than to determine an automatic variable-selection procedure for the final model. Given the number of POAF events and to reduce the risk of overfitting, a reduced multivariable logistic regression model was constructed according to event-per-variable considerations and clinical relevance. In the total cohort, the reduced multivariable model included the TyG index, age, sex, diabetes mellitus, and left atrial diameter. In the non-diabetic subgroup, the reduced multivariable model included the TyG index, age, sex, and left atrial diameter. The TyG index was modeled as a continuous variable. ORs with 95% CIs were reported for all frequentist logistic regression models. To further assess the stability of the association between the TyG index and POAF under regularization, Bayesian logistic regression was performed as a sensitivity analysis. Weakly informative normal priors were assigned to the regression coefficients [N (0, 2.5^2^)], and a weakly informative prior was assigned to the intercept [N (0, 10^2^)]. Posterior ORs with 95% credible intervals were reported. The posterior distribution was summarized using a Laplace approximation. Restricted cubic spline analysis was used to examine the dose-response relationship between the TyG index and POAF. Four knots were placed at the 5th, 35th, 65th, and 95th percentiles of the TyG distribution. The spline model was adjusted for the same variables as the corresponding reduced multivariable model. The median TyG value was used as the reference point. Overall association and non-linearity *P* values were reported. The spline results were interpreted cautiously at the extremes of the TyG distribution because of the smaller number of observations in those ranges. Model discrimination was assessed using receiver operating characteristic (ROC) curve analysis. Rather than evaluating the TyG index as a standalone clinical tool, ROC analyses compared a baseline clinical model with an extended model that additionally included the TyG index. In the total cohort, the baseline clinical model included age, sex, diabetes mellitus, and left atrial diameter, and the extended model additionally included the TyG index. In the non-diabetic subgroup, the baseline clinical model included age, sex, and left atrial diameter, and the extended model additionally included the TyG index. Apparent area under the curve (AUC) values with 95% CIs were reported. Internal validation was performed using bootstrap resampling with 300 repetitions. Optimism-corrected AUC values were calculated to estimate internally validated discrimination performance. Calibration was assessed using calibration plots, the Brier score, and the Hosmer–Lemeshow goodness-of-fit test based on deciles of predicted risk. The incremental value of adding the TyG index to the baseline clinical model was further evaluated using continuous net reclassification improvement (NRI) and integrated discrimination improvement (IDI). As an exploratory analysis to assess whether the TyG index was associated specifically with POAF or reflected a broader tendency toward postoperative complications, crude logistic regression analyses were performed for selected postoperative outcomes, including pneumonia, sepsis, and anastomotic leakage. Because of the limited number of events for sepsis and anastomotic leakage, multivariable adjustment was not performed for these exploratory outcomes. All statistical analyses were performed using R software, version 4.5.2. A two-sided *P* value < 0.05 was considered statistically significant.

## 3 Results

### 3.1 Study population and comparison with patients excluded because of neoadjuvant therapy

Between January 2022 and April 2026, a total of 671 patients were assessed for eligibility. After excluding 117 patients who received preoperative neoadjuvant therapy, 554 patients were included in the primary analytic cohort. Among them, 76 patients developed POAF and 478 patients did not. After excluding 65 patients with diabetes mellitus, 489 patients were included in the non-diabetic subgroup, including 63 patients with POAF and 426 patients without POAF. The study flow diagram is shown in Figure 1. To evaluate the potential selection bias associated with excluding patients who received neoadjuvant therapy, baseline characteristics were compared between the included analytic cohort and the excluded patients. Compared with included patients, patients excluded because of neoadjuvant therapy were younger (61.1 ± 7.2 vs. 64.3 ± 7.9 years; *P* < 0.001), had lower albumin levels (39.2 ± 2.3 vs. 41.2 ± 2.3 g/L; *P* < 0.001), greater weight loss within 3 months before surgery [2.9% (1.3, 5.6) vs. 2.3% (1.1, 4.2); *P* = 0.034], and more advanced clinical TNM stage (*P* < 0.001). However, the TyG index was similar between the two groups (8.46 ± 0.33 vs. 8.49 ± 0.38; *P* = 0.443), and the incidence of POAF did not differ significantly between excluded and included patients (12.8% vs. 13.7%; *P* = 0.797) (Table 1).

**Table 1:** Comparison of included patients and patients excluded due to preoperative neoadjuvant therapy.

Characteristic	Included analytic cohort (*n* = 554)	Excluded due to neoadjuvant therapy (*n* = 117)	*P* value
Age, years	64.3 ± 7.9	61.1 ± 7.2	<0.001
Male sex, *n* (%)	371 (67.0)	84 (71.8)	0.310
TyG index	8.49 ± 0.38	8.46 ± 0.33	0.443
Albumin, g/L	41.2 ± 2.3	39.2 ± 2.3	<0.001
Weight loss within 3 months, %	2.3 (1.1, 4.2)	2.9 (1.3, 5.6)	0.034
POAF, *n* (%)	76 (13.7)	15 (12.8)	0.797
Clinical TNM stage, *n* (%)	554	117	<0.001
I	192 (34.7)	5 (4.3)	
II	331 (59.7)	29 (24.8)	
III	31 (5.6)	83 (70.9)	

Data are presented as mean ± standard deviation, median (interquartile range), or number (percentage), as appropriate. Comparisons were performed using the independent-samples *t* test, Mann–Whitney U test, chi-square test, or Fisher's exact test, as appropriate.

### 3.2 Baseline characteristics according to POAF status in the total cohort

Baseline characteristics of the primary analytic cohort stratified by POAF status are presented in Table 2. The overall incidence of POAF was 13.7% (76/554). Patients who developed POAF had a significantly higher preoperative TyG index than those without POAF (8.70 ± 0.35 vs. 8.45 ± 0.38; *P* < 0.001). They were also older (68.0 ± 7.2 vs. 63.7 ± 7.9 years; *P* < 0.001). The distribution of clinical TNM stage differed between patients with and without POAF (*P* = 0.005), with a higher proportion of stage III disease in the POAF group than in the non-POAF group (13.2% vs. 4.4%). Weight loss within 3 months before surgery [2.8% (1.5, 4.2) vs. 2.2% (1.0, 3.5); *P* = 0.051] and left atrial diameter (38.2 ± 2.8 vs. 37.6 ± 2.8 mm; *P* = 0.086) showed borderline between-group differences. Other baseline and perioperative variables, including sex, BMI, smoking, drinking, hypertension, diabetes mellitus, coronary heart disease, albumin, fasting status confirmation, statin use, metformin use, operative duration, and intraoperative blood loss, were not significantly different between the two groups.

**Table 2:** Baseline characteristics of the analytic cohort stratified by postoperative atrial fibrillation (POAF).

Characteristic	Total (*n* = 554)	No POAF (*n* = 478)	POAF (*n* = 76)	*P* value
TyG index	8.49 ± 0.38	8.45 ± 0.38	8.70 ± 0.35	< 0.001
Age, years	64.3 ± 7.9	63.7 ± 7.9	68.0 ± 7.2	< 0.001
Male sex, *n* (%)	371 (67.0)	322 (67.4)	49 (64.5)	0.619
BMI, kg/m^2^	22.4 ± 2.4	22.4 ± 2.4	22.0 ± 2.4	0.206
Smoking, *n* (%)	216 (39.0)	191 (40.0)	25 (32.9)	0.241
Drinking, *n* (%)	186 (33.6)	165 (34.5)	21 (27.6)	0.238
Hypertension, *n* (%)	180 (32.5)	152 (31.8)	28 (36.8)	0.383
Diabetes mellitus, *n* (%)	65 (11.7)	52 (10.9)	13 (17.1)	0.117
Coronary heart disease, *n* (%)	89 (16.1)	76 (15.9)	13 (17.1)	0.790
Albumin, g/L	41.2 ± 2.3	41.2 ± 2.3	41.1 ± 2.5	0.845
Weight loss within 3 months, %	2.3 (1.1, 4.2)	2.2 (1.0, 3.5)	2.8 (1.5, 4.2)	0.051
Fasting status confirmed, *n* (%)	505 (91.2)	439 (91.8)	66 (86.8)	0.154
Statin use, *n* (%)	35 (6.3)	29 (6.1)	6 (7.9)	0.609
Metformin use, *n* (%)	39 (7.0)	31 (6.5)	8 (10.5)	0.201
Left atrial diameter, mm	37.6 ± 2.9	37.6 ± 2.8	38.2 ± 2.8	0.086
Operative duration, min	260.2 ± 37.9	259.9 ± 37.7	261.8 ± 39.3	0.696
Intraoperative blood loss, mL	119.5 ± 44.8	120.3 ± 44.2	114.5 ± 48.5	0.326
Clinical TNM stage, *n* (%)				0.005
I	192 (34.7)	172 (36.0)	20 (26.3)	
II	331 (59.7)	285 (59.6)	46 (60.5)	
III	31 (5.6)	21 (4.4)	10 (13.2)	

Data are presented as mean ± standard deviation, median (interquartile range), or number (percentage), as appropriate. Comparisons between the No POAF and POAF groups were performed using the independent-samples *t* test, Mann–Whitney U test, chi-square test, or Fisher's exact test, as appropriate.

### 3.3 Univariable associations with POAF

In exploratory univariable logistic regression analyses of the total cohort, a higher TyG index was associated with increased odds of POAF (OR 5.94, 95% CI 2.99–11.83; *P* < 0.001). Age was also associated with POAF (OR 1.07, 95% CI 1.04–1.11; *P* < 0.001). Compared with clinical stage I disease, clinical stage III disease was associated with higher odds of POAF (OR 4.10, 95% CI 1.39–9.08; *P* = 0.002), whereas clinical stage II disease was not significantly associated with POAF. Left atrial diameter showed a borderline association with POAF (OR 1.08, 95% CI 0.99–1.18; *P* = 0.085). The full univariable results are shown in Supplementary Table S2.

### 3.4 Reduced multivariable logistic regression and Bayesian sensitivity analysis in the total cohort

In the reduced multivariable logistic regression model including the TyG index, age, sex, diabetes mellitus, and left atrial diameter, the TyG index remained associated with POAF (OR 4.02, 95% CI 1.90–8.48; *P* < 0.001) (Table 3). Age also remained associated with POAF after adjustment (OR 1.05, 95% CI 1.01–1.09; *P* = 0.007). Male sex, diabetes mellitus, and left atrial diameter were not independently associated with POAF in the reduced model. Bayesian sensitivity analysis yielded directionally consistent results. The posterior OR for the association between the TyG index and POAF was 3.31 (95% credible interval [CrI] 1.64–6.70). The posterior estimate for age was also consistent with the frequentist model (posterior OR 1.05, 95% CrI 1.01–1.09). These findings supported the stability of the association between the TyG index and POAF under a regularized estimation framework.

**Table 3:** Reduced multivariable logistic regression and Bayesian sensitivity analysis for postoperative atrial fibrillation (POAF) in the total cohort.

Variable	Crude model OR (95% CI) *P* value	Reduced multivariable model OR (95% CI) *P* value	Bayesian sensitivity analysis Posterior OR (95% CrI)
TyG index	5.94 (2.99–11.83) *P* <0.001	4.02 (1.90–8.48) *P* <0.001	3.31 (1.64–6.70)
Age, years	1.07 (1.04–1.11) *P* <0.001	1.05 (1.01–1.09) *P* = 0.007	1.05 (1.01–1.09)
Male sex	0.88 (0.53–1.46) *P* = 0.619	0.86 (0.50–1.46) *P* = 0.575	0.85 (0.50–1.44)
Diabetes mellitus	1.69 (0.87–3.28) *P* = 0.121	1.36 (0.67–2.74) *P* = 0.393	1.39 (0.70–2.77)
Left atrial diameter, mm	1.08 (0.99–1.18) *P* = 0.085	1.02 (0.93–1.12) *P* = 0.628	1.01 (0.93–1.11)

Data are presented as odds ratios (ORs) with 95% confidence intervals (CIs) or 95% credible intervals (CrIs), as appropriate. The reduced multivariable model included TyG index, age, sex, diabetes mellitus, and left atrial diameter. For binary variables, ORs compare male vs. female sex and diabetes mellitus vs. no diabetes mellitus. Bayesian sensitivity analysis used weakly informative normal priors on log-odds [coefficients ~ N (0, 2.5^2^); intercept ~ N (0, 10^2^)] and was summarized using a Laplace approximation to the posterior distribution.

### 3.5 Model discrimination, internal validation, calibration, and incremental value of TyG

**Figure 2:**
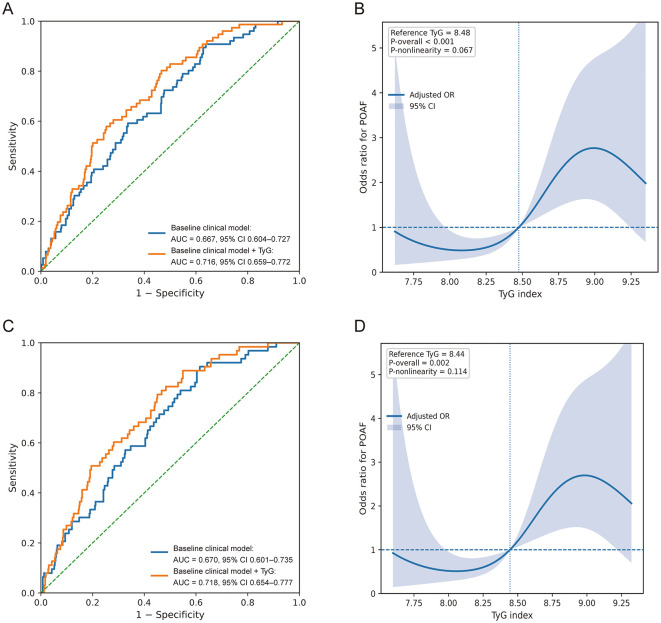
Discrimination and dose-response analyses of the TyG index for POAF. **(A)** ROC curves comparing the baseline clinical model and the extended model including TyG in the total cohort. The baseline model included age, sex, diabetes mellitus, and left atrial diameter; the extended model additionally included TyG. The apparent AUC increased from 0.667 to 0.716 after adding TyG. **(B)** Restricted cubic spline analysis of the association between TyG and POAF in the total cohort, adjusted for age, sex, diabetes mellitus, and left atrial diameter. Four knots were used, and the median TyG value of 8.48 was set as the reference. The overall association was significant (*P*-overall < 0.001), with no statistically significant evidence of non-linearity (*P*-non-linearity = 0.067). **(C)** ROC curves comparing the baseline clinical model and the extended model including TyG in the non-diabetic subgroup. The baseline model included age, sex, and left atrial diameter; the extended model additionally included TyG. The apparent AUC increased from 0.670 to 0.718 after adding TyG. **(D)** Restricted cubic spline analysis of the association between TyG and POAF in the non-diabetic subgroup, adjusted for age, sex, and left atrial diameter. Four knots were used, and the median TyG value of 8.44 was set as the reference. The overall association was significant (*P*-overall = 0.002), with no statistically significant evidence of non-linearity (*P*-non-linearity = 0.114).

**Figure 3:**
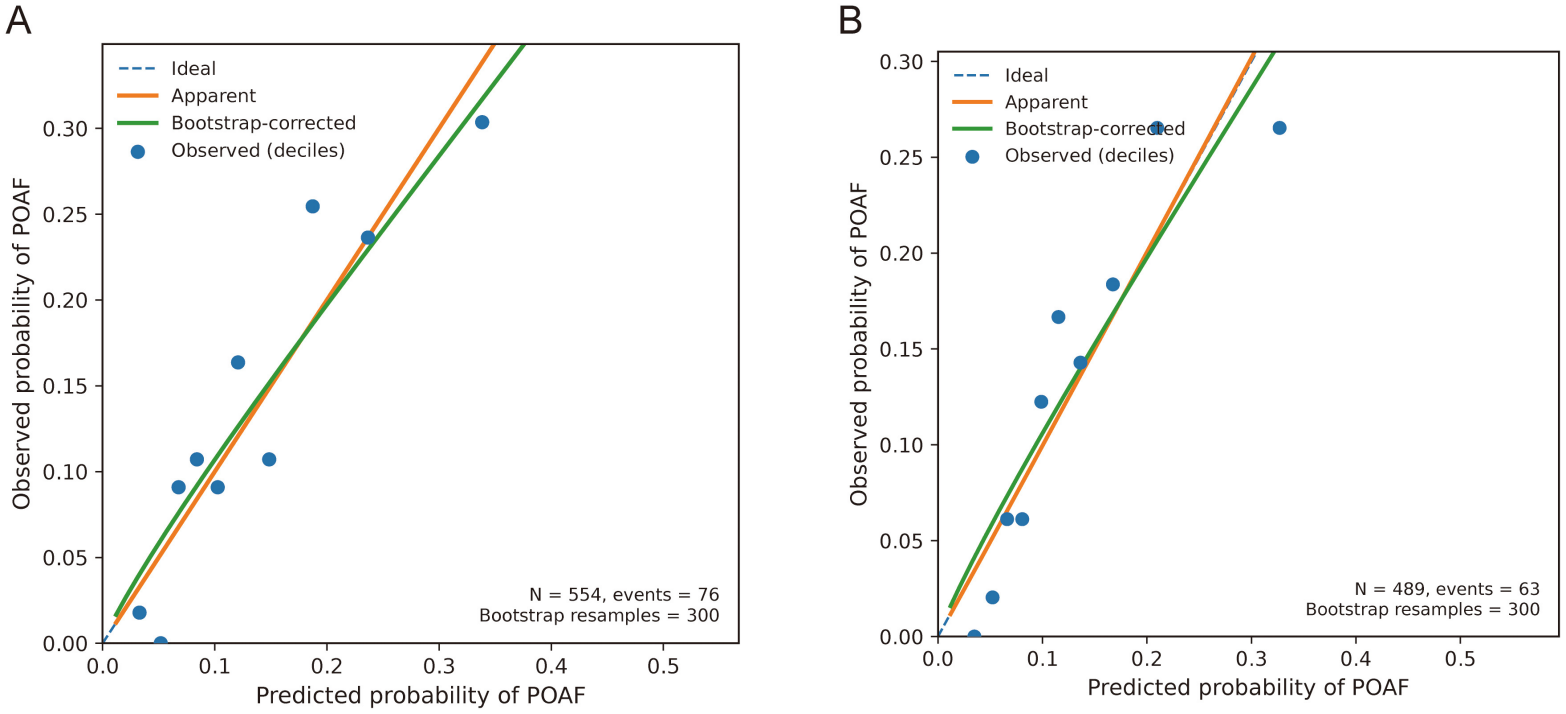
Calibration plots for the extended models including TyG. **(A)** Calibration plot for the extended model in the total cohort, including age, sex, diabetes mellitus, left atrial diameter, and TyG. The total cohort included 554 patients and 76 POAF events. **(B)** Calibration plot for the extended model in the non-diabetic subgroup, including age, sex, left atrial diameter, and TyG. The non-diabetic subgroup included 489 patients and 63 POAF events. Calibration was assessed using 300 bootstrap resamples. The ideal line represents perfect calibration, the apparent line represents calibration in the original dataset, and the bootstrap-corrected line represents internally validated calibration.

Model performance and internal validation results are summarized in Table 4. In the total cohort, the baseline clinical model including age, sex, diabetes mellitus, and left atrial diameter had an apparent AUC of 0.667 (95% CI 0.604–0.727). After adding the TyG index, the apparent AUC increased to 0.716 (95% CI 0.659–0.772) (Figure 2A). Bootstrap internal validation showed that the optimism-corrected AUC increased from 0.647 for the baseline model to 0.694 for the extended model including TyG. The Brier score decreased from 0.114 in the baseline model to 0.111 in the extended model. The Hosmer–Lemeshow test did not indicate poor calibration for either model, with a *P* value of 0.435 for the extended model. Calibration plots based on 300 bootstrap resamples showed broadly acceptable agreement between predicted and observed POAF probabilities, although some variability was observed at higher predicted probabilities (Figure 3A). Addition of the TyG index to the baseline clinical model yielded a continuous NRI of 0.441 and an IDI of 0.024, suggesting modest incremental predictive information beyond the selected clinical variables.

**Table 4:** Model performance, internal validation, and incremental value of adding the TyG index to the baseline clinical model.

Cohort	Model	Apparent AUC	Optimism-corrected AUC	Brier score	Hosmer-Lemeshow *P*	Continuous NRI	IDI
Total cohort	Baseline clinical model	0.667	0.647	0.114	0.628	—	—
Total cohort	Baseline clinical model + TyG	0.716	0.694	0.111	0.435	0.441	0.024
Non-diabetic subgroup	Baseline clinical model	0.670	0.656	0.108	0.414	—	—
Non-diabetic subgroup	Baseline clinical model + TyG	0.718	0.698	0.106	0.598	0.404	0.021

Apparent area under the curve (AUC) values were calculated in the original dataset. Optimism-corrected AUC values were estimated using bootstrap internal validation with 300 resamples. In the total cohort, the baseline clinical model included age, sex, diabetes mellitus, and left atrial diameter; the extended model additionally included the TyG index. In the non-diabetic subgroup, the baseline clinical model included age, sex, and left atrial diameter; the extended model additionally included the TyG index. Hosmer-Lemeshow *P* values were calculated using deciles of predicted risk. Continuous net reclassification improvement (NRI) and integrated discrimination improvement (IDI) quantify the incremental value of adding the TyG index to the baseline model.

### 3.6 Dose-response relationship between the TyG index and POAF

Restricted cubic spline analysis was performed to evaluate the dose-response relationship between the TyG index and POAF. In the total cohort, the spline analysis showed a positive overall association between the TyG index and POAF (*P* for overall association < 0.001). There was no statistically significant evidence of non-linearity, although the non-linearity test showed a borderline pattern (*P* for non-linearity = 0.067). The reference TyG value was 8.48. Confidence intervals widened at the extremes of the TyG distribution, indicating lower precision in these ranges (Figure 2B).

### 3.7 Findings in the non-diabetic subgroup

Baseline characteristics of the non-diabetic subgroup are shown in Supplementary Table S1. Among 489 patients without diabetes mellitus, 63 developed POAF, corresponding to an incidence of 12.9%. Patients with POAF had a higher TyG index than those without POAF (8.67 ± 0.37 vs. 8.43 ± 0.38; *P* < 0.001) and were older (68.2 ± 7.1 vs. 63.8 ± 8.0 years; *P* < 0.001). Clinical TNM stage also differed between the two groups (*P* = 0.012). Left atrial diameter showed a borderline difference (38.17 ± 2.72 vs. 37.50 ± 2.82 mm; *P* = 0.071). In univariable logistic regression, the TyG index was associated with POAF in the non-diabetic subgroup (OR 5.27, 95% CI 2.55–10.91; *P* < 0.001), as was age (OR 1.07, 95% CI 1.04–1.11; *P* < 0.001). Clinical stage III disease was also associated with higher odds of POAF compared with stage I disease (OR 4.11, 95% CI 1.34–8.49; *P* = 0.004). The full univariable results are presented in Supplementary Table S3. In the reduced multivariable model including the TyG index, age, sex, and left atrial diameter, the TyG index remained associated with POAF (OR 3.65, 95% CI 1.66–8.02; *P* = 0.001) (Table 5). Age also remained associated with POAF (OR 1.05, 95% CI 1.01–1.09; *P* = 0.022), whereas sex and left atrial diameter were not independently associated with POAF. Bayesian sensitivity analysis again showed a consistent association between the TyG index and POAF, with a posterior OR of 2.98 (95% CrI 1.42–6.25). In the non-diabetic subgroup, adding the TyG index to the baseline clinical model increased the apparent AUC from 0.670 (95% CI 0.601–0.735) to 0.718 (95% CI 0.654–0.777) (Figure 2C). The optimism-corrected AUC increased from 0.656 to 0.698. The Brier score decreased from 0.108 to 0.106, and the Hosmer–Lemeshow *P* value for the extended model was 0.598. Continuous NRI and IDI were 0.404 and 0.021, respectively (Table 4). Calibration plots based on 300 bootstrap resamples are shown in Figure 3B. Restricted cubic spline analysis in the non-diabetic subgroup showed a positive overall association between the TyG index and POAF (*P* for overall association = 0.002), with no statistically significant evidence of non-linearity (*P* for non-linearity = 0.114). The reference TyG value was 8.44. As in the total cohort, the confidence intervals widened at the extremes of the TyG distribution (Figure 2D).

**Table 5:** Reduced multivariable logistic regression and Bayesian sensitivity analysis in the non-diabetic subgroup.

Variable	Crude model OR (95% CI) *P* value	Reduced multivariable model OR (95% CI) *P* value	Bayesian sensitivity analysis Posterior OR (95% CrI)
TyG index	5.27 (2.55–10.91) *P* < 0.001	3.65 (1.66–8.02) *P* = 0.001	2.98 (1.42–6.25)
Age, years	1.07 (1.04–1.11) *P* < 0.001	1.05 (1.01–1.09) P = 0.022	1.05 (1.01–1.09)
Male sex	0.73 (0.42–1.26) *P* = 0.260	0.67 (0.38–1.18) *P* = 0.166	0.67 (0.38–1.18)
Left atrial diameter, mm	1.09 (0.99–1.20) *P* = 0.077	1.04 (0.94–1.15) *P* = 0.426	1.03 (0.93–1.14)

Data are presented as odds ratios (ORs) with 95% confidence intervals (CIs) or 95% credible intervals (CrIs), as appropriate. The non-diabetic subgroup included patients without a clinical diagnosis of diabetes mellitus at baseline (*n* = 489). The reduced multivariable model included TyG index, age, sex, and left atrial diameter. For binary variables, ORs compare male vs. female sex. Bayesian sensitivity analysis used weakly informative normal priors on log-odds [coefficients ~ N (0, 2.5^2^); intercept ~ N (0, 10^2^)] and was summarized using a Laplace approximation to the posterior distribution.

### 3.8 Exploratory analyses of other postoperative outcomes

To explore whether the TyG index was associated specifically with POAF or reflected a broader tendency toward postoperative complications, crude associations between the TyG index and selected postoperative outcomes were examined in the total cohort. In addition to POAF, postoperative pneumonia occurred in 59 patients (10.6%), sepsis in 18 patients (3.2%), and anastomotic leakage in 14 patients (2.5%). The TyG index was significantly associated with POAF in crude analysis (OR 5.94, 95% CI 2.99–11.83; *P* < 0.001), but it was not significantly associated with pneumonia (OR 1.75, 95% CI 0.86–3.55; *P* = 0.123), sepsis (OR 1.13, 95% CI 0.33–3.86; *P* = 0.843), or anastomotic leakage (OR 2.02, 95% CI 0.50–8.12; *P* = 0.321) (Supplementary Table S4). These analyses were exploratory and unadjusted because of the limited number of events for these postoperative outcomes.

## 4 Discussion

In this retrospective cohort of patients undergoing curative esophagectomy for esophageal squamous cell carcinoma, a higher preoperative TyG index was associated with increased odds of POAF. This association persisted after adjustment for selected clinically relevant covariates in a reduced multivariable model and remained directionally consistent in Bayesian sensitivity analysis. Similar findings were observed in the non-diabetic subgroup, suggesting that the association between TyG and POAF was not solely driven by patients with clinically diagnosed diabetes mellitus. In addition, adding TyG to a baseline clinical model modestly improved discrimination, internally validated performance, and reclassification indices. Restricted cubic spline analysis suggested a positive overall association between TyG and POAF without statistically significant evidence of non-linearity, although the estimates were less precise at the extremes of the TyG distribution. Taken together, these findings indicate that the preoperative TyG index may provide complementary cardiometabolic information for POAF risk assessment after esophagectomy (15).

A major consideration in studies of perioperative risk prediction is the balance between confounding control and model stability. In the present analysis, we used a parsimonious modeling strategy based on event-per-variable considerations and clinical relevance, in line with contemporary recommendations for reducing overfitting in clinical prediction modeling (16). Rather than constructing extensively adjusted models with a high risk of overfitting, the primary multivariable model included TyG, age, sex, diabetes mellitus, and left atrial diameter in the total cohort, and TyG, age, sex, and left atrial diameter in the non-diabetic subgroup. This strategy was intended to preserve model stability while adjusting for key clinical factors related to atrial arrhythmia risk. The association between TyG and POAF was attenuated but remained evident in Bayesian sensitivity analysis using weakly informative priors, supporting the stability of the estimate under a regularized framework. Furthermore, the predictive contribution of TyG was evaluated using not only apparent AUC but also bootstrap optimism-corrected AUC, calibration assessment, Brier score, continuous NRI, and IDI, consistent with recommendations that prediction models should be evaluated across complementary performance domains rather than by discrimination alone (17, 18). These analyses provide a more balanced assessment of the incremental value of TyG than discrimination alone.

From a clinical perspective, the TyG index is attractive because it is derived from routinely available fasting triglyceride and fasting plasma glucose measurements and has been increasingly recognized as a practical cardiometabolic biomarker (8, 15). Unlike insulin-based measures of insulin resistance, TyG can be calculated without additional specialized testing and may therefore be easily incorporated into preoperative assessment (7, 19). However, the observed improvement in model performance should be interpreted as modest rather than definitive. In the total cohort, adding TyG to the baseline clinical model improved the optimism-corrected AUC, and similar findings were observed in the non-diabetic subgroup. Nevertheless, these results do not support the use of TyG as a standalone tool for predicting POAF or selecting prophylactic antiarrhythmic interventions. Instead, TyG should be viewed as a potentially useful complementary marker that may add a cardiometabolic dimension to conventional risk assessment based on age, diabetes status, and atrial structural features (2, 6, 13).

The persistence of the association in patients without diabetes mellitus is an important finding. Diabetes is a recognized risk factor for atrial fibrillation (20), but diabetes status alone may not fully capture the spectrum of cardiometabolic heterogeneity (14). Some patients without overt diabetes may still have unfavorable glucose-lipid profiles or subclinical metabolic abnormalities (15, 21). In this context, TyG may help identify a subgroup of non-diabetic patients with an unfavorable cardiometabolic profile and increased perioperative vulnerability to atrial arrhythmia (22). However, this interpretation should remain cautious. Because insulin, C-peptide, and HOMA-IR were not available in this study, we cannot confirm that TyG directly reflected insulin resistance in this cohort. Therefore, the present findings should be interpreted as demonstrating an association between a routinely available cardiometabolic marker and POAF, rather than proving a causal role of insulin resistance in postoperative arrhythmogenesis.

Although the mechanisms underlying the association between TyG and POAF were not directly examined, several plausible pathways may be considered based on prior work linking TyG to cardiometabolic dysfunction and POAF to perioperative inflammatory and autonomic stress (6, 23, 24). Higher TyG values may reflect an unfavorable metabolic milieu characterized by impaired glucose-lipid homeostasis, which has been linked to endothelial dysfunction, oxidative stress, inflammatory activation, and myocardial metabolic vulnerability (25, 26). These processes may contribute to atrial structural and electrical instability, particularly under the physiological stress of major thoracic surgery. Esophagectomy is associated with substantial surgical trauma, autonomic perturbation, fluid shifts, and systemic inflammatory responses, all of which may lower the threshold for atrial arrhythmias in susceptible patients (27–29). Nevertheless, these mechanistic interpretations remain hypothesis-generating because inflammatory biomarkers, insulin-based metabolic indices, and direct measures of atrial substrate were not available in the present study.

The restricted cubic spline analyses provided additional information on the exposure-response pattern. In both the total cohort and the non-diabetic subgroup, TyG showed a significant positive overall association with POAF. There was no statistically significant evidence of non-linearity, although the total cohort showed a borderline non-linear pattern. This finding should not be interpreted as evidence of a definitive threshold. Confidence intervals widened at the lower and upper ends of the TyG distribution, indicating reduced precision in those regions. Therefore, the present data do not support a clinically actionable TyG cut-off for POAF risk stratification, a cautious interpretation consistent with recent reviews emphasizing the context-specific nature and limitations of TyG-based risk assessment (15, 30). Any threshold-based application of TyG would require validation in independent cohorts and, ideally, prospective studies with standardized perioperative monitoring, in line with contemporary recommendations for clinical prediction model evaluation and external validation (31, 32).

We also explored whether the observed association between TyG and POAF might simply reflect a broader tendency toward postoperative complications, given that pneumonia, anastomotic leakage, atrial arrhythmias, and other complications frequently occur after esophagectomy and may be clinically interrelated (3, 33, 34). In crude exploratory analyses, TyG was significantly associated with POAF but was not significantly associated with pneumonia, sepsis, or anastomotic leakage. These findings provide some reassurance that the TyG-POAF association may not merely represent a non-specific marker of global postoperative vulnerability. However, these analyses should be interpreted cautiously because the number of events for sepsis and anastomotic leakage was limited, and multivariable adjustment was not performed for these outcomes. Therefore, the specificity of TyG for POAF remains incompletely established and should be examined in larger cohorts with broader postoperative outcome assessment.

The exclusion of patients who received preoperative neoadjuvant therapy also deserves careful consideration. Neoadjuvant chemotherapy, immunotherapy, or targeted therapy may influence nutritional status, body composition, systemic inflammatory or immune status, tumor stage, and perioperative risk (35–37). Excluding these patients reduced treatment-related heterogeneity in the primary analytic cohort, but it also limits the generalizability of the findings to contemporary patients with locally advanced esophageal cancer, for whom neoadjuvant or multimodal therapy is commonly used (38, 39). In the present study, patients excluded because of neoadjuvant therapy differed from included patients in age, nutritional indicators, and clinical stage, although TyG and POAF incidence were not significantly different between the groups (Table 1). These findings suggest that selection bias cannot be fully excluded, and the applicability of the present results to patients receiving neoadjuvant therapy requires further investigation.

Several additional limitations should be acknowledged. First, this was a single-center retrospective study, and residual confounding cannot be eliminated despite multivariable adjustment and sensitivity analyses. Second, although the reduced modeling strategy was chosen to minimize overfitting, the findings still require external validation in independent cohorts. Internal validation by bootstrap resampling can estimate optimism within the current dataset, but it cannot establish generalizability across institutions, surgical techniques, or perioperative care pathways. Third, fasting status could not be confirmed in all patients, which may have introduced measurement variability in triglyceride and glucose values. Fourth, although statin and metformin use were collected, other medication exposures and perioperative management factors may not have been fully captured. Fifth, nutritional assessment was limited. Albumin and recent weight loss were available, but more detailed measures such as prealbumin, skeletal muscle index, CT-defined sarcopenia, and comprehensive nutritional scoring were not included. This is particularly relevant in esophageal cancer, where malnutrition and body composition changes are common and may influence both metabolic markers and postoperative outcomes. Sixth, asymptomatic or short-lived atrial arrhythmias before surgery may not have been completely excluded, and postoperative POAF episodes after transfer from the intensive care unit may have been missed if they were clinically silent. Finally, the exploratory analyses of pneumonia, sepsis, and anastomotic leakage were unadjusted because of limited event numbers, and therefore cannot definitively establish outcome specificity.

In conclusion, a higher preoperative TyG index was associated with increased odds of POAF after esophagectomy, including among patients without diabetes mellitus. TyG provided modest incremental predictive information beyond selected clinical factors and may serve as a readily available complementary marker in perioperative risk assessment. However, the present findings do not support the use of TyG as a standalone predictive tool or as the basis for threshold-based clinical decision-making. Prospective multicenter studies with external validation, standardized rhythm monitoring, detailed metabolic and nutritional assessment, and broader postoperative outcome evaluation are needed before TyG-guided risk stratification or intervention strategies can be recommended.

## Data Availability

The original contributions presented in the study are included in the article/Supplementary material, further inquiries can be directed to the corresponding authors.
