# Supplementary material for: Association of the preoperative triglyceride-glucose index with postoperative atrial fibrillation following esophagectomy: a retrospective cohort study with a focus on non-diabetic patients
Source: Front Nutr. 2026 May 29;13:1785056. doi: 10.3389/fnut.2026.1785056 (PMC13261753; doi:10.3389/fnut.2026.1785056)
Supplement: Supplementary file 1 [file Supplementary_file_1.docx]

Supplementary Table S1. Baseline characteristics of the non-diabetic subgroup stratified by postoperative atrial fibrillation (POAF).

| Characteristic | Total  (n = 489) | No POAF  (n = 426) | POAF  (n = 63) | P value |
| --- | --- | --- | --- | --- |
| TyG index | 8.46 ± 0.38 | 8.43 ± 0.38 | 8.67 ± 0.37 | <0.001 |
| Age, years | 64.4 ± 8.0 | 63.8 ± 8.0 | 68.2 ± 7.1 | <0.001 |
| Male sex, n (%) | 333 (68.1) | 294 (69.0) | 39 (61.9) | 0.258 |
| BMI, kg/m² | 22.26 ± 2.41 | 22.33 ± 2.41 | 21.82 ± 2.36 | 0.119 |
| Smoking, n (%) | 185 (37.8) | 164 (38.5) | 21 (33.3) | 0.430 |
| Drinking, n (%) | 169 (34.6) | 151 (35.4) | 18 (28.6) | 0.284 |
| Hypertension, n (%) | 155 (31.7) | 134 (31.5) | 21 (33.3) | 0.765 |
| Coronary heart disease, n (%) | 75 (15.3) | 62 (14.6) | 13 (20.6) | 0.211 |
| Fasting status confirmed, n (%) | 447 (91.4) | 392 (92.0) | 55 (87.3) | 0.212 |
| Statin use, n (%) | 26 (5.3) | 22 (5.2) | 4 (6.3) | 0.761 |
| Albumin, g/L | 41.18 ± 2.34 | 41.18 ± 2.31 | 41.14 ± 2.50 | 0.904 |
| Weight loss within 3 months, % | 2.1 (1.2, 3.8) | 2.0 (1.2, 3.5) | 2.5 (1.5, 3.8) | 0.112 |
| Clinical TNM stage, n (%) |  |  |  | 0.012 |
| I | 156 (31.9) | 140 (32.9) | 16 (25.4) |  |
| II | 313 (64.0) | 273 (64.0) | 40 (63.5) |  |
| III | 20 (4.1) | 13 (3.1) | 7 (11.1) |  |
| Left atrial diameter, mm | 37.58 ± 2.81 | 37.50 ± 2.82 | 38.17 ± 2.72 | 0.071 |
| Operative duration, min | 259.3 ± 38.2 | 258.9 ± 37.9 | 261.8 ± 40.7 | 0.598 |
| Intraoperative blood loss, mL | 119.4 ± 44.1 | 120.7 ± 43.9 | 110.2 ± 44.6 | 0.084 |

Footnote: Data are presented as mean ± standard deviation, median (interquartile range), or number (percentage), as appropriate. Comparisons between the No POAF and POAF groups were performed using the independent-samples t test, Mann–Whitney U test, chi-square test, or Fisher’s exact test, as appropriate.

Supplementary Table S2. Univariable logistic regression analyses for postoperative atrial fibrillation (POAF) in the total cohort.

| Variable | OR (95% CI) | P value |
| --- | --- | --- |
| TyG index | 5.94 (2.99–11.83) | <0.001 |
| Age, years | 1.07 (1.04–1.11) | <0.001 |
| Male sex | 0.88 (0.53–1.46) | 0.619 |
| BMI, kg/m² | 0.94 (0.85–1.04) | 0.205 |
| Smoking | 0.74 (0.44–1.23) | 0.242 |
| Drinking | 0.72 (0.42–1.24) | 0.239 |
| Hypertension | 1.25 (0.76–2.07) | 0.384 |
| Diabetes mellitus | 1.69 (0.87–3.28) | 0.121 |
| Coronary heart disease | 1.09 (0.57–2.08) | 0.790 |
| Fasting status confirmed | 0.59 (0.28–1.23) | 0.158 |
| Statin use | 1.33 (0.53–3.31) | 0.544 |
| Metformin use | 1.70 (0.75–3.84) | 0.205 |
| Albumin, g/L | 0.99 (0.89–1.10) | 0.837 |
| Weight loss within 3 months, % | 1.02 (0.95–1.09) | 0.577 |
| Clinical stage II vs I | 1.39 (0.79–2.43) | 0.249 |
| Clinical stage III vs I | 4.10 (1.39–9.08) | 0.002 |
| Left atrial diameter, mm | 1.08 (0.99–1.18) | 0.085 |
| Operative duration, min | 1.00 (0.99–1.01) | 0.686 |
| Intraoperative blood loss, mL | 1.00 (0.99–1.00) | 0.292 |

Footnote: Odds ratios (ORs) are from separate univariable logistic regression models in the analytic cohort (n = 554). For binary variables, ORs compare the presence versus absence of the characteristic or male versus female sex. Clinical TNM stage comparisons use stage I as the reference group.

Supplementary Table S3. Univariable logistic regression analyses for postoperative atrial fibrillation (POAF) in the non-diabetic subgroup.

| Variable | OR (95% CI) | P value |
| --- | --- | --- |
| TyG index | 5.27 (2.55–10.91) | <0.001 |
| Age, years | 1.07 (1.04–1.11) | <0.001 |
| Male sex | 0.73 (0.42–1.26) | 0.260 |
| BMI, kg/m² | 0.92 (0.82–1.02) | 0.121 |
| Smoking | 0.80 (0.46–1.40) | 0.431 |
| Drinking | 0.73 (0.41–1.30) | 0.286 |
| Hypertension | 1.09 (0.62–1.91) | 0.765 |
| Coronary heart disease | 1.53 (0.78–2.97) | 0.214 |
| Fasting status confirmed | 0.60 (0.26–1.35) | 0.217 |
| Statin use | 1.24 (0.41–3.74) | 0.696 |
| Albumin, g/L | 0.99 (0.89–1.11) | 0.898 |
| Weight loss within 3 months, % | 1.00 (0.93–1.08) | 0.939 |
| Clinical stage II vs I | 1.28 (0.69–2.31) | 0.428 |
| Clinical stage III vs I | 4.11 (1.34–8.49) | 0.004 |
| Left atrial diameter, mm | 1.09 (0.99–1.20) | 0.077 |
| Operative duration, min | 1.00 (1.00–1.01) | 0.576 |
| Intraoperative blood loss, mL | 0.99 (0.99–1.00) | 0.078 |

Footnote: Odds ratios (ORs) are from separate univariable logistic regression models in the non-diabetic subgroup (n = 489). For binary variables, ORs compare the presence versus absence of the characteristic or male versus female sex. Clinical TNM stage comparisons use stage I as the reference group.

Supplementary Table S4. Exploratory crude associations between the TyG index and postoperative outcomes in the total cohort.

| Outcome | Events, n (%) | Crude OR for TyG  (95% CI) | P value |
| --- | --- | --- | --- |
| POAF | 76 (13.7) | 5.94 (2.99–11.83) | <0.001 |
| Pneumonia | 59 (10.6) | 1.75 (0.86–3.55) | 0.123 |
| Sepsis | 18 (3.2) | 1.13 (0.33–3.86) | 0.843 |
| Anastomotic leakage | 14 (2.5) | 2.02 (0.50–8.12) | 0.321 |

Footnote: Odds ratios (ORs) are from crude logistic regression models using the TyG index as a continuous predictor. The analyses for pneumonia, sepsis, and anastomotic leakage are exploratory and were intended to assess whether the TyG index was associated with competing postoperative outcomes beyond POAF. Because of the limited number of sepsis and anastomotic leakage events, multivariable models for those outcomes were not pursued in this supplementary analysis.
